# Identification of Antimicrobial Metabolites from the Egyptian Soil-Derived *Amycolatopsis keratiniphila* Revealed by Untargeted Metabolomics and Molecular Docking

**DOI:** 10.3390/metabo13050620

**Published:** 2023-04-30

**Authors:** Ahmed A. Hamed, Osama G. Mohamed, Elsayed A. Aboutabl, Fify I. Fathy, Ghada A. Fawzy, Riham A. El-Shiekh, Ahmed A. Al-Karmalawy, Areej M. Al-Taweel, Ashootosh Tripathi, Tarek R. Elsayed

**Affiliations:** 1 Pharmacognosy Department, Faculty of Pharmacy, Cairo University, Kasr el Aini St., Cairo 11562, Egypt; 2 Natural Products Discovery Core, Life Sciences Institute, University of Michigan, Ann Arbor, MI 48109, USA; 3 Pharmaceutical Chemistry Department, Faculty of Pharmacy, Ahram Canadian University, 6th of October City, Giza 12566, Egypt; 4 Department of Pharmacognosy, College of Pharmacy, King Saud University, Riyadh 11495, Saudi Arabia; 5 Department of Medicinal Chemistry, College of Pharmacy, University of Michigan, Ann Arbor, MI 48109, USA; 6 Agricultural Microbiology Department, Faculty of Agriculture, Cairo University, Giza 12613, Egypt

**Keywords:** actinomycetes, *Amycolatopsis*, antibiotics, metabolomics, Egyptian soil, molecular docking, ECO-0501

## Abstract

Actinomycetes are prolific producers of bioactive secondary metabolites. The prevalence of multidrug-resistant (MDR) pathogens has prompted us to search for potential natural antimicrobial agents. Herein, we report the isolation of rare actinobacteria from Egyptian soil. The strain was identified as *Amycolatopsis keratiniphila* DPA04 using 16S rRNA gene sequencing. Cultivation profiling, followed by chemical and antimicrobial evaluation of crude extracts, revealed the activity of DPA04 ISP-2 and M1 culture extracts against Gram-positive bacteria. Minimum inhibitory concentrations (MIC) values ranged from 19.5 to 39 µg/mL. Chemical analysis of the crude extracts using ultra-high performance liquid chromatography-quadrupole time-of-flight mass spectrometry (UHPLC-QTOF) led to the identification of 45 metabolites of different chemical classes. In addition, ECO-0501 was identified in the cultures with significant antimicrobial activity. Multidrug resistance in *Staphylococcus aureus* is reported to be related to the multidrug efflux pump (MATE). ECO-0501 and its related metabolites were subjected to molecular docking studies against the MATE receptor as a proposed mechanism of action. ECO-0501 and its derivatives (AK_1 and *N-*demethyl ECO-0501) had better binding scores (−12.93, −12.24, and −11.92 kcal/mol) than the co-crystallized 4HY inhibitor (−8.99 kcal/mol) making them promising candidates as MATE inhibitors. Finally, our work established that natural products from this strain could be useful therapeutic tools for controlling infectious diseases.

## 1. Introduction

Antibiotics are considered one of the most important discoveries of the 20th century used to treat infectious diseases. The combined efforts of science in the past century have led to the introduction of different classes of effective antibiotics [1]. However, pathogenic microbes continuously evolve and adapt over time to available antibiotics, leading to antimicrobial resistance (AMR). AMR spreads from one bacterium to another through mobile genetic elements such as interferons, plasmids, transposons, and bacteriophages. The spread of multidrug-resistant (MDR) pathogens is a significant problem for global health, making it challenging to use antibiotics effectively [2,3,4]. The World Health Organization (WHO) has placed AMR on its list of the top 10 threats to global public health. According to a report from the WHO, AMR kills approximately 700,000 people worldwide every year and is predicted to kill 10 million people annually by 2050 [5].

Actinomycetes are one of the most abundant sources of new antibiotics, such as glycopeptides, macrolides, aminoglycosides, rifamycins, tetracyclines and β-lactams [6,7,8]. Collections of these organisms were previously based on random screening, with limited knowledge of the diversity of microorganisms in the sampled material. Recent research has focused on rare actinomycetes to support the market with novel potential antibiotics. Approximately 25% of antibiotics originated from rare actinomycetes. Some genera in this group are *Amycolatopsis*, *Nocardia*, *Actinomadura*, *Actinoplanes*, *Ampullariella*, *Actinosynnema*, *Dactylosporangium*, and *Microbiospora* [9].

*Amycolatopsis* strains produce various secondary metabolites, including polyphenols, linear polyketides, macrolides, sesquiterpenes, thiazolyl peptides, cyclic peptides, amide derivatives, enediyne derivatives, glycoside derivatives, and macrolactams [10]. Most of these are essential antibiotics, including rifamycin and vancomycin. Due to its potential medicinal applications, further research on the genus *Amycolatopsis* should be considered. Numerous parameters should be considered when studying how a strain produces secondary metabolites. For example, the composition of the medium, pH, temperature, amount of oxygen available, and light intensity can affect the metabolism of microbes and, consequently, the production of compounds [11]. The OSMAC (One Strain–Many Compounds) approach uses various culture conditions to elicit the expression of different metabolites. OSMAC may activate silent genes, which increases the chances of finding novel metabolites [12].

Right now, the discovery of known compounds is one of the major challenges in natural products research. Therefore, it is crucial to annotate compounds quickly before they are isolated. Metabolomics based on mass spectrometry (MS) has been growingly used because of its sensitivity and versatility. However, MS can produce many spectra, which makes analysis challenging [13]. Molecular networking using the Global Natural Products Social Molecular Networking (GNPS) web-based platform has proven its applicability in analyzing large sets of MS data. In addition, many tools available on GNPS can conduct an automated search for spectral matches aided by public spectral libraries [14,15]. In addition, the chemical environment within a metabolomics experiment can be assessed using the molecular networking tool provided by (GNPS) [16].

Therefore, the primary purposes of this work were to explore the influence of varying culture conditions on the secondary metabolites production of terrestrial *Amycolatopsis keratiniphila* DPA04 and tentatively identify the antibacterial metabolites produced by this strain using Liquid Chromatography with tandem mass spectrometry (LC-MS/MS), which was evaluated utilizing computational chemistry using molecular docking to discover, propose, or recommend a definite mechanism of action for identified compounds.

## 2. Experimental

### 2.1. Isolation of A. keratiniphila DPA04

Soil samples were obtained from the Agricultural Experiment and Research Station of Cairo University. Soil samples weighing less than 200 g each were taken by inserting a spatula, sterilized with ethyl alcohol (70%), into the sediments. The soil samples were transferred to polybags, sealed tightly, and immediately transported to the lab [17]. The samples were left at room temperature for one week to dry in the air and were then stored at 4 °C. Using the dilution plate method, serial dilutions were prepared from the soil samples, and actinomycetes were isolated on starch-nitrate agar (SNA) plates. To stop the growth of fungal species and Gram-negative bacteria, 50 mg/mL cycloheximide and 20 mg/mL nalidixic acid were added to the SNA medium. The plates were incubated for seven days at 30 °C. Colonies with typical Actinomycete morphology were selected, and their purity was examined by repeated subculturing. For further use, pure cultures were kept in Luria-Bertani (LB) broth with 20% glycerol at −20 °C [18].

### 2.2. 16S rRNA Gene Sequencing and Phylogenic Analysis of A. keratiniphila DPA04 Strain

Using the Bio-Rad T100 thermal cycler and the universal primers F-27 (5′-AGAGTTTGATCMTGGCTCAG-3′) and R1494 (5′-CTACGGYTACCTTGTTACGAC-3′) [19,20], *A. keratiniphila’*s 16S rRNA gene fragments were amplified. The initial step of PCR, the denaturation, was carried out at 95 °C for 12 min. Then, 30 cycles of 94 °C for 1 min, 56 °C for 1 min, and 72 °C for 2 min. At the end of the PCR reaction, there was one extension step at 72 °C for 10 min. Five microliters of PCR products were processed via electrophoresis on 1% agarose gel in 0.5x TBE-buffer for one hour (80 V). Gels were stained with ethidium bromide, DNA was detected under UV light, purified with a gel extraction kit, and then sequenced by Macrogen (Seoul, Republic of Korea). Phylogenetic analysis was carried out using the neighbor-joining method with Maximum Composite Likelihood [21]. This was undertaken by comparing the sequences of the 16S ribosomal RNA (16S rRNA) gene amplified from the bacterial isolate in this study with the most similar hits from the National Center for Biotechnology Information (NCBI) GenBank database using BLASTn (http://blast.ncbi.nlm.nih.gov/Blast.cgi (accessed on 21 January 2023)) [22]. Evolutionary analyses were studied by the MEGA 5 software [23]. The 16S rRNA gene sequence of the DPA04 isolate was uploaded to the NCBI GenBank database under the accession number (OP970553).

### 2.3. Preparation of Crude Extracts

*A. keratiniphila* DPA04 was inoculated on two individual plates from the following culture media. Starch nitrate agar (SNA) medium, ISP-2 agar medium, M1 agar medium, R2YE agar medium, and Czapek agar medium and kept at 30 °C for 7 days. The composition of the medium is listed in Table S1. Following incubation, the agar cultures were chopped into 1.5 cm × 1.5 cm pieces, transferred to 250 mL flasks, and extracted with ethyl acetate (EtOAc) (2 × 80 mL) overnight at 150 rpm. Using a rotatory evaporator at 40 °C, the decanted organic layer (supernatant) was evaporated under vacuum to yield the crude extract. The dry extract was dissolved in methanol (MeOH), then prepared to have a concentration of 1 mg/mL, and filtered on a 13 mm diameter and 0.2 µm in pores membrane (VWR International). An aliquot (30 µL) was kept for LC-MS/MS analysis. To evaluate the antimicrobial activity, the remaining extracts were evaporated and redissolved in 20% (*v*/*v*) Dimethyl sulfoxide (DMSO) at a 2.5 mg/mL concentration. Non-inoculated media agar plates were prepared the same way as negative controls for LC-MS/MS analysis and antimicrobial activity.

### 2.4. Antibacterial Activity

The bacterial extracts were tested against *E. coli* 0157 H:7, Methicillin-resistant *Staphylococcus aureus* (MRSA) ATCC 43300, *Staphylococcus aureus* ATCC 6538P, *Salmonella typhimurium* ATCC 14028, *Listeria monocytogenes* ATCC 19115, *Bacillus cereus* ATCC 33,018 and *Pseudomonas aeruginosa* ATCC 9027. A modified agar-well diffusion method was used to test the antimicrobial activity [24]. In total, 1.0 mL of the standard suspension (5 × 10^5^ CFU/mL) of each test strain of bacteria was evenly distributed on Mueller Hinton Agar plates with a sterile glass rod spreader and allowed to dry at room temperature. Then, 6 mm agar wells were formed, and 100 μL of the bacterial extract reconstituted in 20% DMSO to 2.5 mg/mL was pipetted into triplicate wells. Following 1 h at room temperature to let the extract disseminate into the agar, the plates were incubated at 37 °C for 24 h, and the inhibitory zone diameter was measured to the nearest millimeter. The antibiotics polymyxin and novobiocin (5 μg/mL) served as positive controls for Gram-negative and Gram-positive bacteria, respectively. Non-inoculated media extracts in (20% *v*/*v*) DMSO were served as the negative control.

### 2.5. Determination of the Minimal Inhibitory Concentration (MIC)

The modified agar-well diffusion method was used to evaluate the MIC [25]. A two-fold serial dilution of each extract in 20% DMSO yielded 0.00975–2.5 mg/mL concentration range. Then, 100-uL dilutions were applied to triplicate wells of Mueller Hinton Agar (MHA) plates pre-inoculated with test bacterial cells. At 37 °C, all of the test plates were kept for 24 h. The minimal inhibitory concentration (MIC) was established by using the concentration of each extract or control that produced a discernible inhibition zone.

### 2.6. Liquid Chromatography with Tandem Mass Spectrometry (LC-MS/MS) Analysis

Ultra-high performance liquid chromatographic (UHPLC) analysis was performed using an Agilent LC-MS system composed of an Agilent 1290 Infinity II UHPLC linked to an Agilent 6545 ESI-Q-TOF-MS according to the method described by Hamed et al. [26], aliquots (1 µL) of EtOAc extract (1 mg/mL in MeOH) were assessed using a Kinetex phenyl-hexyl (1.7 μm, 2.1 × 50 mm) column. With a flow rate of 0.4 mL/min, the column was eluted with 1 min isocratic elution of 90% A (A: 100% water (H_2_O) + 0.1% formic acid) followed by 6 min linear gradient elution to 100% B (95% Acetonitrile (MeCN) + 5% H_2_O + 0.1% formic acid). Electrospray ionization (ESI) settings were 11 L/min sheath gas flow, 3.5 kV source voltage and 320 °C capillary temperature. The entire scan detected ions over 1000 counts at six scans/s, ramping collision energy (5 × m/z/100 + 10 eV), a maximum of nine selected precursors per cycle, with an isolation width of 1.3 ~*m*/*z*. For negative mode, the internal lock masses, hexakis (1H,1H,3H-tetrafluoropropoxy)-phosphazene (C_18_H_18_F_24_N_3_O_6_P_3_) [M+TFA−H]^−^ ion (*m*/*z* 1033.9881) and Trifluoroacetic acid (TFA,C_2_HF_3_O_2_) [M−H]^−^ ion (*m*/*z* 112.9856) were employed. For positive mode, the internal lock masses, hexakis (C_18_H_18_F_24_N_3_O_6_P_3_) [M+H] + ion (*m*/*z* 922.0098) and purine (C_5_H_4_N_4_) [M+H]^+^ ion (*m*/*z* 121.0509) were employed.

### 2.7. MS/MS Data Preprocessing

Msconvert proteoWizard tool was used to convert raw data files to mzXML format [27]. mzXML files were processed using MZmine 2.53 to create feature peak lists (for the parameters, see Table S2) [28]. Features corresponding to blank media and MeOH were removed.

### 2.8. Feature-Based Molecular Networking

For molecular network construction, the Feature-Based Molecular Networking (FBMN) workflow on The GNPS was employed [16]. The cross-platform Winscp was used to upload the mgf files to the GNPS server. The precursor and fragment ion mass tolerances were set to 0.02 Da. Advanced network presets: minimum pair cosine:0.65 for positive mode data, 0.7 for negative mode and minimum matching fragment ions of four. The library search employed a score cutoff of 0.65 and minimum matching peaks of four. The default values were set for all other parameters and Cytoscape 3.9.1 generated the molecular network [29].

### 2.9. Molecular Formula Prediction and Metabolites Identification

The metabolites identification was accomplished by thorough investigation of the high-resolution mass spectra. Sirius 5.5.7, based on high-resolution mass spectrometry, determined each analyte’s elemental composition [30]. The analyte’s most likely elemental composition was determined via accurate mass and fragmentation patterns and searched in chemical structure databases. Molecular annotation was conducted using SIRIUS 5.5.7’s CSI: FingerID interface [31]. Natural products atlas [32], molDiscovery [33], and Reaxys [34]. The list of metabolites that fitted the examined molecular formula were carefully checked for being bacterial metabolites. The mass error of all identified metabolites was below 10 ppm.

### 2.10. Molecular Docking Studies

ECO-0501 and its related derivatives were subjected to molecular docking studies against the multidrug efflux pump (MATE) receptor (PDB ID: 5C6O) [35]. The Molecular Operating Environment (MOE) 2019.0102 software [36,37] was used to compare the binding affinities of the investigated compounds to the co-crystallized native inhibitor (4YH) of the target protein. Therefore, the 4YH of the MATE receptor was placed into the prepared database as a reference standard. The metabolites were sketched using the ChemDraw software and introduced separately into the MOE working window, where their partial charges were corrected. Subsequently, the energy is minimized [38,39]. All prepared compounds were transferred into a single database with a co-crystallized inhibitor (4YH). The target MATE protein receptor was obtained from the Protein Data Bank website (PDB ID:5C6O) [35], opened with the MOE, corrected for the missed parts, 3D hydrogenated, and energy minimized, as described previously [40,41,42]. Finally, a general docking process was performed using the aforementioned databases to compare the binding affinities of the investigated compounds and 4YH inhibitor of the target MATE protein. The program specifications were adjusted according to the default parameters [43,44].

## 3. Results

### 3.1. Taxonomic Identification of A. keratiniphila DPA04

BLAST analysis of the NCBI database showed that the strain with the code DPA04 had the closest 16s rRNA gene sequence to *A. keratiniphila* (99 to 100% identity) using Maximum Likelihood analysis (Figure S1). The MEGA 5 software was used to generate the phylogenetic tree.

### 3.2. Antibacterial Activity

The crude extracts of *A. keratiniphila* DPA04 were investigated for antibacterial activity against *E. coli 0157 H:7*, *MRSA ATCC 43300*, *S. aureus ATCC 6538P*, *S. typhimurium ATCC 14028*, *L. monocytogenes ATCC 7494*, *P. aeruginosa ATCC 9027*, and *B. cereus ATCC 33018*, exhibiting different susceptibility responses, as shown in Table 1.

Only two of the DPA04 crude extracts, M1 and ISP2 cultures, had strong antibacterial activity against Gram-positive bacteria with zones of inhibitions range (22–32 mm) for ISP-2 culture extract and (19–27 mm) for M1 culture extract. Table 2 displays the MIC values for the active cultures ranging from 19.5 to 39 μg/mL.

### 3.3. Chemical Analysis

**Figure 1:**
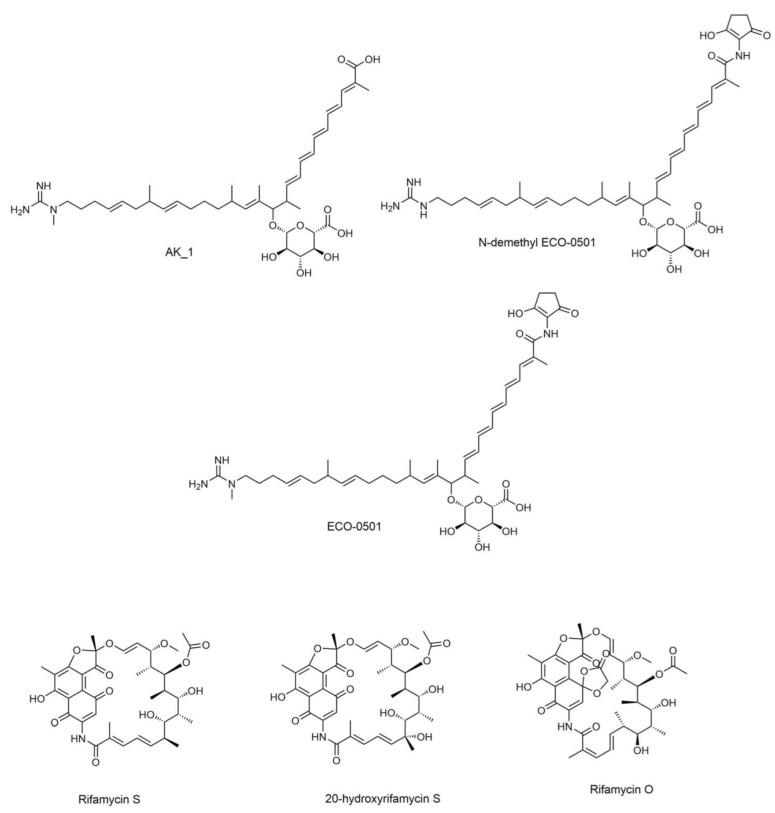
Antibacterial metabolites annotated in DPA04 EtOAc extracts.

Metabolite profiling was accomplished using UHPLC-ESI-QTOF-MS/MS-based molecular networking to discover promising candidates with antimicrobial activity. The identification was based on a literature review, GNPS library search [16], SIRIUS platform [27], and other platforms such as Reaxys [34] and NP Atlas [29]. Many detected metabolites have been previously reported to have several biological activities [30,31,32,33]. The selected structures of the metabolites with antibacterial activity are shown in Figure 1. The analysis led to the identification of 45 secondary metabolites.

The identified compounds are listed in Table S3, while antibacterial metabolites identified in DPA04 EtOAc extracts are listed in Table 3. Various metabolites have been identified from actinomycetes and classified into sugars, meroterpenoids, macrolides, peptides, glycosidic polyketides, phospholipids, and siderophores. They were previously reported as unique anti-cancer, antibacterial, antifungal, antioxidant, and antidiabetic compounds [45,46,47,48,49,50,51,52,53,54].

Visualization of MS/MS data through molecular networking makes it possible to annotate metabolites and highlight differences between different samples, which in this study are different culturing media of the DPA04 strain. Each node in the network corresponds to one consensus MS/MS spectrum that shows the mass of the precursor ion (*m*/*z*). The edges connect the nodes with similar fragmentation spectra. The node color denotes the culture medium and the edge label denotes the cosine score. These nodes are shown as pie charts, showing the relative quantity of each ion in the examined extracts. The ionization modes of the investigated bacterial strain are demonstrated to have two distinct molecular networks: one for positive mode (Figure S2) and one for negative mode (Figure S3).

**Figure 2:**
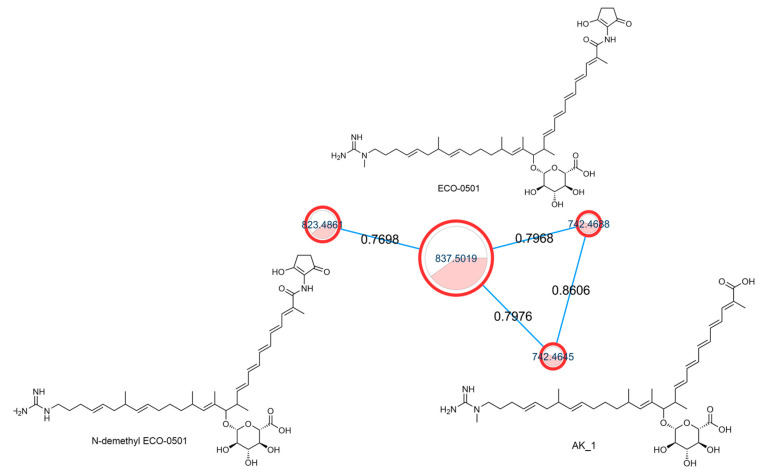
ECO-0501 spectral family with structures of annotated metabolites where the node color represents culturing media as follows: white = ISP-2, red = M1, while the edge label denotes the cosine score.

Among the identified secondary metabolites, ECO-0501 was identified in the active extracts. ECO-0501 was isolated from *Amycolatopsis orientalis* ATCC 43491. ECO-0501 has potent antibacterial activity against several Gram-positive pathogens, including vancomycin-resistant enterococci (VRE) and MRSA [55]. Figure 2 shows the ECO-0501 cluster, which contains related metabolites.

**Figure 3:**
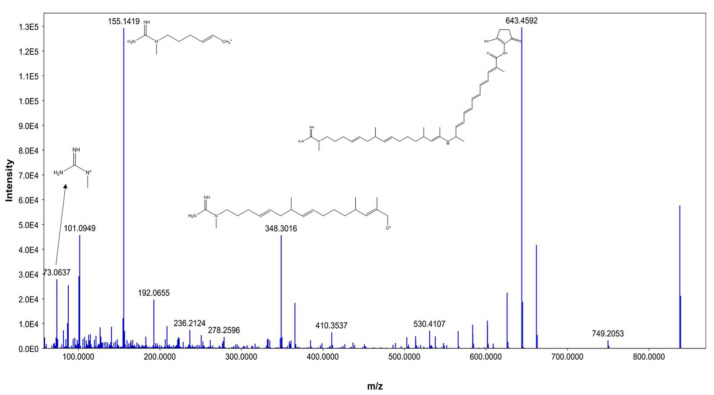
MS/MS fragmentation of ECO-0501.

From our data, the molecular ion peaks of [M+H]^+^ at *m*/*z* 837.5019 and [M-H]^−^ at *m*/*z* 835.4863, both of which match ECO-0501 (Compound 6), were detected in the LC-HR-MS spectra of DPA04 grown on M1 and ISP-2 media. Regarding structure, ECO-0501 is a glycosidic polyketide with a polyketide chain comprising an uncommon combination of aminohydroxycyclopentenone, glucuronic acid, and guanidine groups. Key fragments such as 643.4592, 348.3016, 155.1419, and 73.0637 represented the loss of glucuronic acid, loss of the alkyl chain connected to the aminohydroxycyclopentenone group, formation of allylic carbocation connected to the methyl guanidine group by (CH_2_)_3_, and methyl guanidinium cation, respectively, as shown in Figure 3.

**Figure 4:**
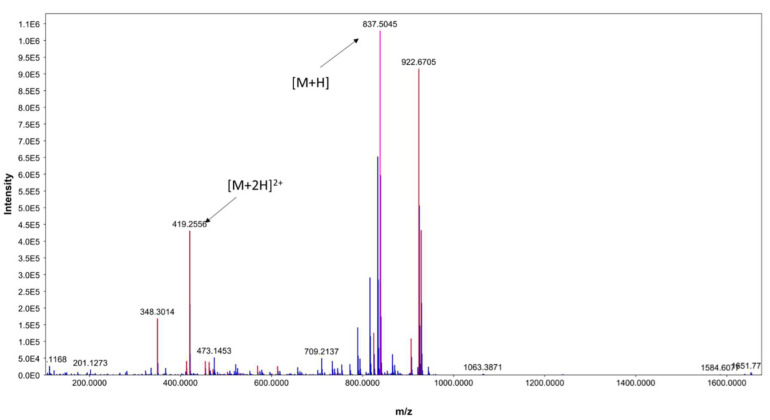
ESI(+)MS spectrum of ECO-0501.

In addition, the existence of [M+2H]^2+^ at *m*/*z* 419.2556 further indicates ECO-0501 presence (Figure 4). Notably, this metabolite was only produced in M1 and ISP-2 media, which showed the highest bioactivity when the DPA04 strain was tested. The bioactivity profile of DPA04 can be explained by the presence of ECO-0501.

**Figure 5:**
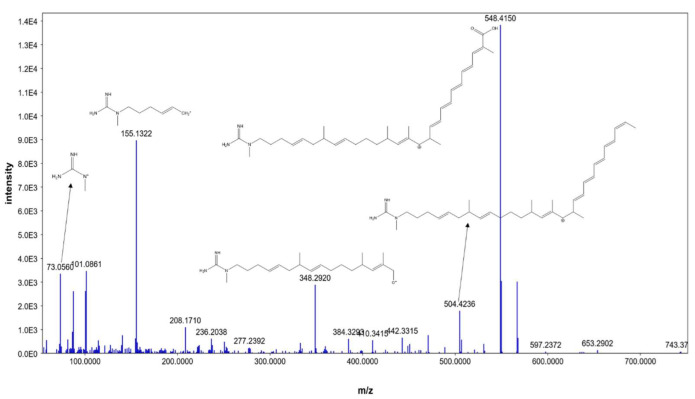
MS/MS fragmentation of AK_1.

Additionally, compound **4** was identified as AK_1 and compound **5** as *N-*demethyl ECO-0501, where both exhibited molecular ion peaks of [M+H]^+^ at *m*/*z* 742.4645 and 835.4863. Key fragments, such as 548.4150, 504.4236, 348.2920, 155.1322, and 73.0560, corresponded with the anticipated fragmentation pattern of AK_1, as shown in Figure 5. AK-1, a precursor of ECO-0501, showed a similar pattern to that of ECO-0501. However, it was devoid of the aminohydroxycyclopentenone group, which a carboxylic terminal group replaced. This could be explained by product ions *at m*/*z* = 548.4150 and 504.4236, representing carboxylic group loss of the aglycone part.

**Figure 6:**
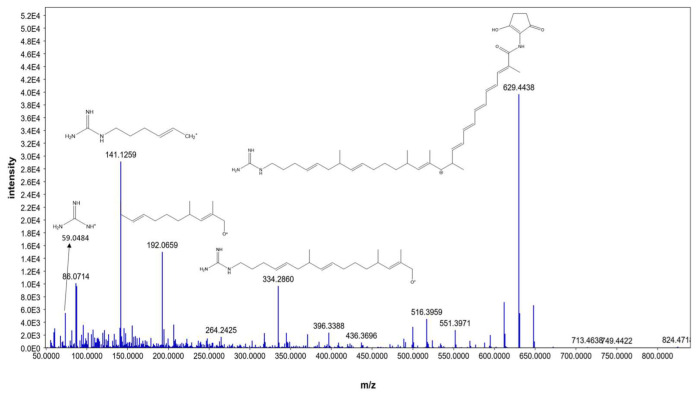
MS/MS fragmentation pattern of *N-*demethyl ECO-0501.

Moreover, *N*-demethyl ECO-0501 exhibited characteristic fragments at 629.4438, 334.2860, 192.0659, 141.1259, and 59.0484, corresponding to the anticipated fragmentation pattern of *N-*demethyl ECO-0501, as shown in Figure 6, *N*-demethyl ECO-0501 showed a pattern similar to that of ECO-0501; however, the fragments were shifted by *m*/*z* = 14 owing to the absence of the *N*-methyl group. To our knowledge, this is the first report of the mass fragmentation of ECO-0501 and its analogs.

### 3.4. Docking Studies

ECO-0501 and its related metabolites were docked against the multidrug efflux pump (MATE) using the MOE 2019.0102 [36]. This was conducted to propose their MATE receptor-targeting antibacterial mode of action. Additionally, the co-crystallized 4YH inhibitor MATE was put into the same database as a reference standard.

The native co-crystallized 4YH inhibitor MATE formed three hydrogen bonds with MET64, MET67, and GLN252. Therefore, these amino acids are crucial for the antagonistic activity against the MATE receptor. It was found that ECO-0501 and its metabolites (AK_1 and *N-*demethyl ECO-0501) were the most promising candidates with superior binding scores (−12.93, −12.24, and −11.92 kcal/mol) compared to the co-crystallized 4HY inhibitor (−8.99 kcal/mol).

The main compound (ECO-0501) formed four hydrogen bonds with MET67, ASN37, and MET296. However, its first metabolite (AK_1) bound to MET67, ASN158, and GLN399 via three hydrogen bonds. On the other hand, its second metabolite (*N-*demethyl ECO-0501) achieved three hydrogen bonds with GLN251, GLN252, and ASP40. Additionally, the docked co-crystallized 4YH inhibitor formed three hydrogen bonds with MET64, MET67, and GLN252, in addition to a hydrogen-pi bond with PHE154 (Table 4).

The above findings recommend the promising and potential antagonistic activities of the identified compounds (ECO-0501, AK_1, and *N-*demethyl ECO-0501) as MATE inhibitors. This can be confirmed through their superior binding scores and interactions with the crucial amino acids of the MATE receptor pocket as well.

## 4. Discussion

Actinomycetes are among the richest providers of secondary metabolites and produce potent bioactive metabolites [56]. The genus *Amycolatopsis* is a significant supplier of many antibiotics and other useful bioactive natural products. Rifamycin and vancomycin are the two most well-known antibiotics *Amycolatopsis* strains produce [10,56]. Recent years have seen a surge in interest in the OSMAC strategy. By using this approach, which entails changing the fermentation conditions of a specific bacterium, the latter’s genetic expression can be boosted, and the latter’s potential as a creator of uncommon bioactive metabolites can be exploited [57]. By applying different culturing conditions, we investigated the antibacterial activity of a rare actinomycete, *A. keratiniphila* DPA04, and tentatively identified the antibacterial metabolites produced by this strain. In our study, *A. keratiniphila* DPA04 M1 and ISP-2 EtOAc extracts showed promising antibacterial activity against Gram-positive bacteria. ECO-0501, a metabolite obtained from *Amycolatopsis orientalis*, was identified in the active extracts and was reported to have potential against Gram-positive pathogens including MDR isolates [55,58]. In addition, we performed mass spectrometry-based identification of forty-five metabolites.

ECO-0501, a structurally unique glycosidic polyketide antibiotic, was identified in the active extracts. It was anticipated that a gene cluster with type I polyketide synthase would mediate the biosynthesis of ECO-0501, which could be considered a novel antibiotic against drug-resistant Gram-positive bacteria [10]. ECO-0501 showed activity against MRSA and VRE (vancomycin-resistant enterococci) [55]. Besides, FBMN along with extensive analysis of fragmentation pattern of ECO-0501 led us to putatively identify two structurally related metabolites (Ak_1 and *N*-demethyl ECO-0501). Due to their close structure similarity, they were proposed to have similar mechanisms of action. Future studies are required to isolate and determine the clinical relevance of these metabolites. Studying these metabolites is required to establish their safety and effectiveness and to develop a structure-activity relationship (SAR) model to improve their pharmacodynamics and pharmacokinetics, which could be followed by animal model testing. The synergistic effects between reported drugs and within these metabolites should be studied more to find out the mechanism beyond their antimicrobial effects.

MATE family transporters, a recently discovered class of drug efflux pumps, are discovered in both Gram-positive and Gram-negative bacteria [59]. Furthermore, multidrug resistance in *Staphylococcus aureus*, a significant issue in hospital infections, is reportedly caused by the multidrug efflux pump (MATE) [43]. MATE transporters could transport structurally dissimilar antibiotics such as ampicillin, kanamycin, norfloxacin, chloramphenicol, and many others making bacteria resistant to these antibiotics [59]. So far, no bacterial efflux pump inhibitors have been approved for treating bacterial infections in humans. More attention should be paid to finding new MATE inhibitors because this group of transporters is growingly detected in MDR strains. ECO-0501 and its related metabolites identified by molecular networking were docked against the multidrug efflux pump (MATE). ECO-0501 and its derivatives (AK_1 and *N*-demethyl ECO-0501) had superior binding scores compared to the co-crystallized 4HY inhibitor. Based on this, we suggest the prospective and possible antagonistic activities of ECO-0501, AK 1, and *N*-demethyl ECO-0501 as MATE inhibitors. In addition, the expected potential antimicrobial activities of the main compound (ECO-0501) and its two metabolites (AK_1 and *N-*demethyl ECO-0501) suggest synergetic use with antimicrobial medications to combat multidrug-resistant strains, resulting in the inclusion of ECO-0501 and its derivatives in the treatment of MDR *Staphylococcus aureus* strains.

Clinical studies have investigated the macrolactam class of metabolites since 1940 [10]. Rifamycins are antibiotics that belong to the ansamycin family. They combat *Mycobacterium tuberculosis* by blocking the bacterial deoxyribonucleic acid (DNA)-dependent ribonucleic acid (RNA) polymerase [10]. On M1 and ISP-2 media, the DPA04 strain produced rifamycin S, 20-hydroxyrifamycin S, and rifamycin O. Rifamycins have several drawbacks, including hepatotoxicity, cytochrome P450 induction, and the rapid selection of resistant mutants [60]. Finding novel rifamycin analogs could boost their effectiveness against mutant strains while decreasing their potential for adverse effects. Only two annotated nodes were in the rifamycin clusters shown in Figure S4, whereas the remaining ones represented unidentified metabolites. Thus, the DPA04 strain may be a natural source for developing new rifamycin derivatives.

It has been demonstrated that the OSMAC technique can stimulate biosynthetic gene clusters (BGCs), making it a potentially useful tool for developing novel bioactive compounds. The OSMAC approach obtained the DPA04 strain to produce bioactive metabolites. The production of some secondary metabolites could be boosted through changing the culture medium (i.e., changing the concentration of carbon, nitrogen, phosphorous, inorganic salts, etc.) [61]. Thus, we used bioassays with the OSMAC technique to select optimal growth conditions for expressing a diverse set of antimicrobial metabolites.

Herein, for the first time, this study reports an extensive chemical investigation of *A. keratiniphila* and its potential production of antibacterial agents using different culture conditions. Further, we explored the MATE-inhibitory of selected metabolites via molecular docking studies. In the future, improvements in the expression of cryptic gene clusters in those *Amycolatopsis* strains will be valuable in acquiring novel metabolites. Comparative genomes, metabolomics, and proteomics in culture-based investigations are uncovering regulation systems, opening new avenues for exploring non-expressed pathways. Moreover, strategies inspired by bioinformatics and functional genomics could be employed to discover the metabolites which are not detected under normal culturing conditions. Further, heterologous expression techniques could be employed to produce novel antimicrobial metabolites and to overcome the slow costly process of large-scale production of actinobacteria secondary metabolites.

## 5. Conclusions

Here, we report the isolation of the rare actinomycete *A. keratiniphila* DPA04 from Egyptian soil samples. The crude extracts, M1 and ISP2 cultures, had strong antibacterial activity against Gram-positive bacteria with MIC values ranging from 19.5 to 39 μg/mL. Using UHPLC-ESI-QTOF-MS/MS-based molecular networking, 45 secondary metabolites were detected. Applying our integrative approach, we determined the optimal culture conditions for producing antimicrobial metabolites. Moreover, ECO-0501-related metabolites (ECO-0501, AK_1, and *N*-demethyl ECO-0501) showed a potential antagonistic activity as MATE inhibitors in the molecular docking study, as reflected from their binding scores (−12.93, −12.24, and −11.92 kcal/mol) compared to the co-crystallized 4HY inhibitor (−8.99 kcal/mol). Future scale-up investigations are required to verify these compounds’ chemical structure and bioactivity profiles. Finally, ECO-0501 is a potent bioactive compound and its derivatives are obtained from *A. keratiniphila*, and their implementation in therapeutics and drug discovery is highly recommended.

## Figures and Tables

**Table 1:** The mean diameter of inhibition zones of the *A. keratiniphila* DPA04 ethyl acetate extracts.

Mean Diameter of Inhibition Zone (MDIZ)/mm							
	SNA	ISP-2	M1	R2YE	Czapek	Positive Control	Negative Control
*Staphylococcus aureus*	-	24	20	-	-	22	-
*Methicillin-resistant Staphylococcus aureus* (MRSA)	-	22	19	-	-	24	-
*Bacillus cereus*	-	24	22	-	-	24	-
*Salmonella typhimurium*	-	-	-	-	-	18	-
*Pseudomonas aeruginosa*	-	-	-	-	-	14	-
*Listeria monocytogenes*	-	32	27	-	-	24	-
*E. coli* O157:H7	-	-	-	-	-	14	-

The antibiotics polymyxin and novobiocin (5 µg/mL) were added as positive controls for Gram-negative and Gram-positive bacteria, respectively. Non-inoculated media agar extract in 20% DMSO was served as the negative control. (-); no activity.

**Table 2:** Minimum inhibitory concentration (MIC) of active ethyl acetate extracts.

Minimal Inhibitory Concentration (MIC, µg/mL)					
	SNA	ISP-2	M1	R2YE	Czapek
*Staphylococcus aureus*	-	39	39	-	-
*Methicillin-resistant Staphylococcus aureus* (MRSA)	-	39	39	-	-
*Bacillus cereus*	-	19.5	19.5	-	-
*Salmonella typhimurium*	-	-	-	-	-
*Pseudomonas aeruginosa*	-	-	-	-	-
*Listeria monocytogenes*	-	19.5	39	-	-
*E. coli* O157:H7	-	-	-	-	-

The data were compared with the negative controls, non-inoculated media agar extracts, in 20% DMSO. (-): no activity.

**Table 3:** A list of annotated antibacterial compounds in *A. keratiniphila* DPA04.

No.	R_t_ (Min)	Compound Name	Adduct	Precursor Mass	Molecular Formula	MS/MS Fragmentation Product Ions	Chemical Class
1	2.90	20-Hydroxyrifamycin S	M-H	711.289094	C_37_H_45_NO_13_	123.045, 180.1031, 221.0471, 253.0712	Macrocyclic lactams
2	3.21	Rifamycin S	M-H	695.294179	C_37_H_45_NO_12_	123.0453, 153.0559, 180.1034, 221.0462	Macrocyclic lactams
3	3.85	Rifamycin O	M-H	753.299659	C_39_H_47_NO_14_	119.0862, 154.0744, 163.0772, 192.1029	Macrocyclic lactams
4	4.13	AK_1	M+H	741.456432	C_41_H_63_N_3_O_9_	73.056, 101.0861, 155.1322, 548.415	Linear polyketides
5	4.41	*N-*demethyl ECO-0501	M+H	822.477896	C_45_H_66_N_4_O_10_	86.0714, 141.1259, 192.0659, 629.4438	Linear polyketides
6	4.48	ECO-0501	M+H	836.493545	C_46_H_68_N_4_O_10_	101.0949, 155.1419, 348.3016, 643.4592	Linear polyketides

**Table 4:** 3D binding interactions and receptor positioning for ECO-0501, AK-1, and *N*-demthyl ECO-0501 and the docked 4YH inhibitor of the MATE receptor pocket.

Compound	3D Interactions	3D Positioning
ECO-0501	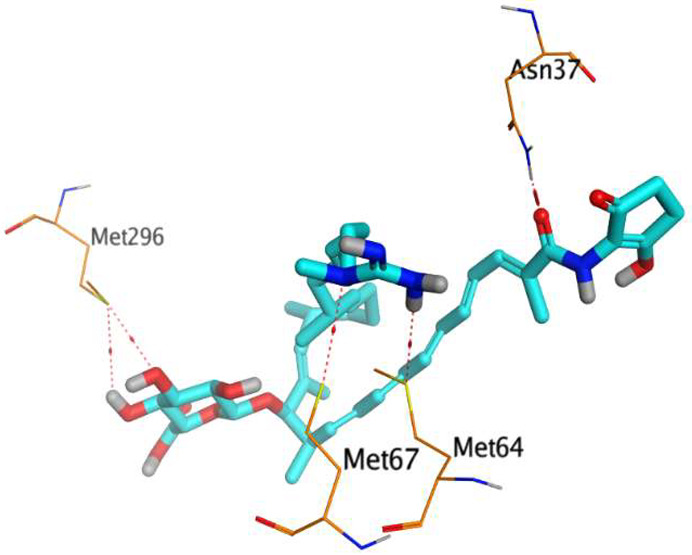	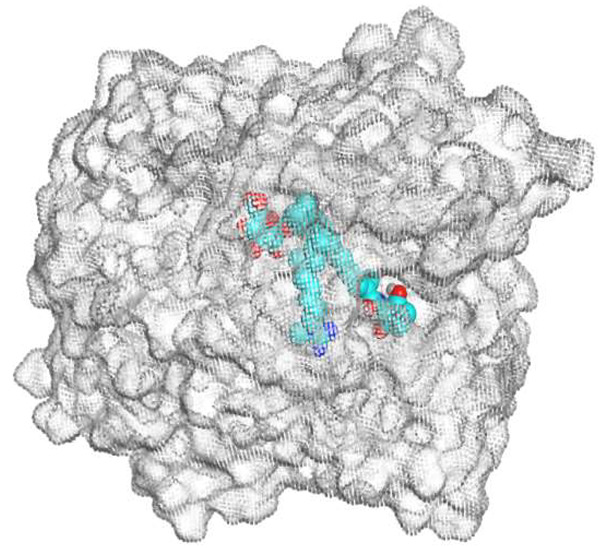
AK_1	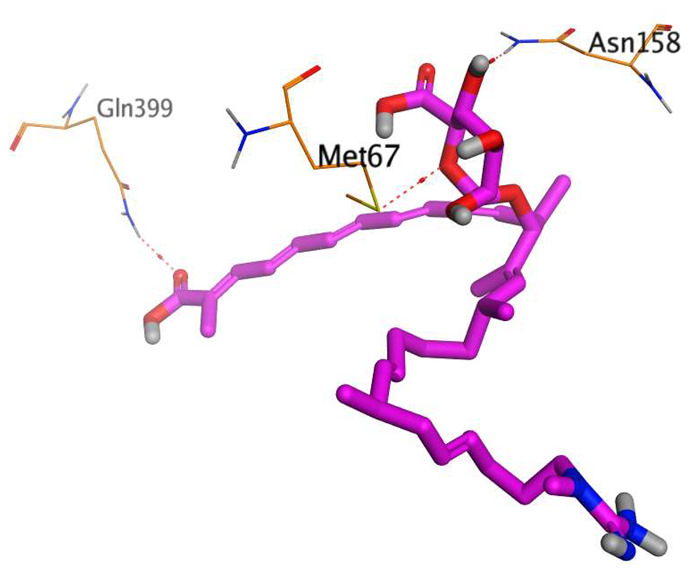	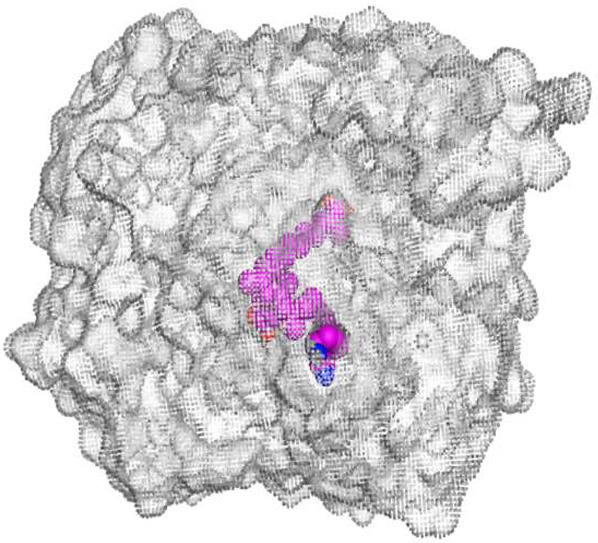
*N-*demethyl ECO-0501	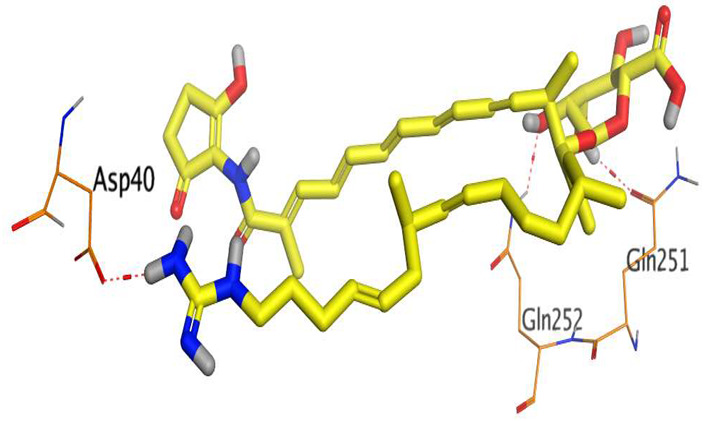	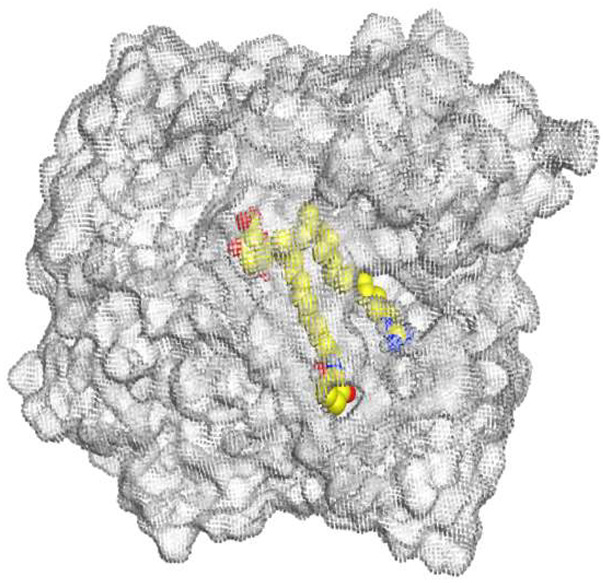
4YH	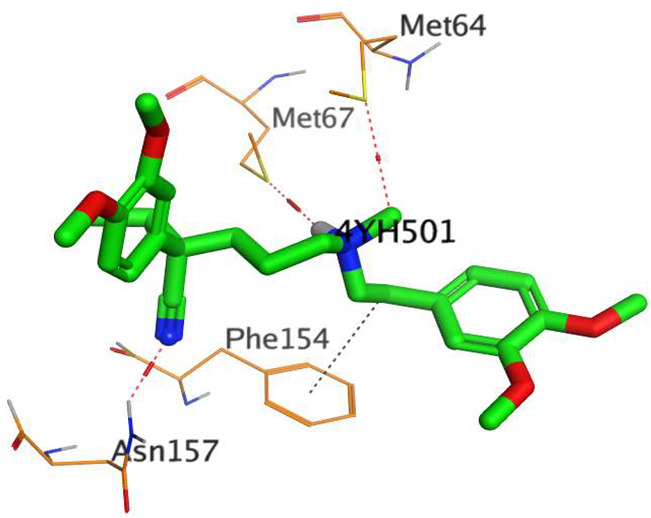	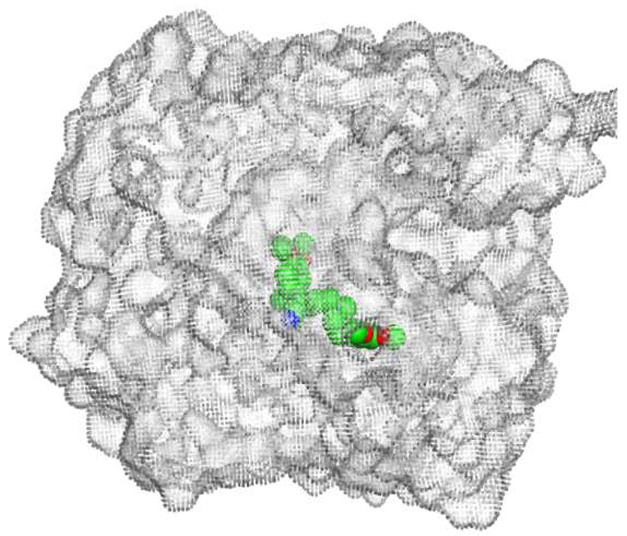

## Data Availability

Not applicable.

## Supplementary Materials

The following supporting information can be downloaded at: https://www.mdpi.com/article/10.3390/metabo13050620/s1, Figure S1. A neighbor-joining phylogenetic tree based on 16s rRNA sequences. Dark circle represents bacterial isolate used in this study DPA04; bootstrap values are indicated at each node; Figure S2. Molecular network of positive ion mode data where the node color represents culture media as follows: white = ISP-2, red = M1, purple = SNA, orange = R2YE, and yellow = Czapek.; Figure S3. Molecular network of negative mode data where the node color represents culturing media as follows: white = ISP-2, red = M1, purple = SNA, orange = R2YE and yellow = Czapek.; Figure S4. Rifamycins spectral family with annotated metabolites where the node color represents culturing media: white = ISP-2, red = M1; The edge label represents the cosine score; Table S1: Types and compositions of solid-based media; Table S2: Parameters for MZmine processing of UHPLC-MS/MS data; Table S3. A list of annotated compounds in *A. keratiniphila* DPA04.
