# Supplementary material for: Identification of Antimicrobial Metabolites from the Egyptian Soil-Derived Amycolatopsis keratiniphila Revealed by Untargeted Metabolomics and Molecular Docking
Source: Metabolites. 2023 Apr 30;13(5):620. doi: 10.3390/metabo13050620 (PMC10221244; doi:10.3390/metabo13050620)
Supplement: Supplementary file 1 [file metabolites-13-00620-s001.zip › metabolites-2286892-supplementary.pdf]

metabolites

MDPI

Supporting Information

Identification of Antimicrobial Metabolites from The Egyptian soil-derived *Amycolatopsis keratiniphila* revealed by Untargeted Metabolomics and Molecular Docking

Ahmed A. Hamed[1*], Osama G. Mohamed[1,2], Elsayed A. Aboutabl[1], Fify I. Fathy[1], Ghada A. Fawzy[1], Riham A. El-Shiekh[1], Ahmed A. Al-Karmalawy[3*], Areej M. Al-Taweel[4], Ashootosh Tripathi[2,5], and Tarek R. Elsayed[6]

[1]Pharmacognosy Department, Faculty of Pharmacy, Cairo University, Kasr el Aini St., Cairo 11562, Egypt.

[2]Natural Products Discovery Core, Life Sciences Institute, University of Michigan, Ann Arbor, MI 48109, USA.

[3]Pharmaceutical Chemistry Department, Faculty of Pharmacy, Ahram Canadian University, 6th of October City, Giza 12566, Egypt.

[4]Department of Pharmacognosy, College of Pharmacy, King Saud University, Riyadh 11495, Saudi Arabia.

[5]Department of Medicinal Chemistry, College of Pharmacy, University of Michigan, Ann Arbor, MI 48109, USA.

[6]Agricultural Microbiology Department, Faculty of Agriculture, Cairo University, Giza 12613, Egypt.

Table S1: Types and compositions of solid-based media

Medium	Composition (per Litre)
SNA	Starch (10.0 g), Casein (0.3 g), KNO_3 (2.0 g), NaCl (2.0 g), K_2HPO_4 (2.0 g), $MgSO_4.7H_2O$ (0.05 g), $CaCO_3$ (0.02 g), $FeSO_4.7H_2O$ (0.01 g), Agar (20.0 g); pH 7.0.
ISP-2	Yeast extract (4.0 g), Malt extract (10.0 g), Dextrose (4.0 g) and Agar (20.0 g); pH 7.2.
M1	Peptone (2.0 g), Yeast extract (4.0 g), Starch (10.0 g) and Agar (20.0 g); pH 7.0.
R2YE	Glucose (10.0 g), K_2SO_4 (0.25 g), $MgCl_2$ (10.12 g), Casamino acid (0.1 g), Yeast extract (5.0 g), 10 mL of 0.5% KH_2PO_4, 80 mL of 3.68% $CaCl_2$, 15 mL of 20% L-proline, 100 mL of 5.73% TES buffer, 2 mL of trace elements solution and Agar (20.0 g); pH 7.2.
Czapek	K_2HPO_4 (1.0 g), $NaNO_3$ (0.3 g), KCl (0.005 g), $MgSO_4.7H_2O$ (0.005 g), $FeSO_4$ (0.0001 g), Sucrose (30 g) and Agar (20.0 g); pH 7.0.

Table S2: Parameters for MZmine processing of UHPLC-MS/MS data

Processing step	Parameter	Selected values
Mass list	MS1 noise level	1.0E3
	MS2 noise level	1.0E2
	Rt (retention time in minutes)	0-7.5 min
Chromatogram building	Algorithm	ADAP chromatogram builder
	Min group size in number of scans	5
	Group intensity threshold	3.0E3
	Min highest intensity	7.0E3
	m/z tolerance	0-20 ppm
Deconvolution	Algorithm	Local minimal search
	Chromatographic threshold	90.0%
	Search minimum in RT range	0.05 min
	Minimum relative height	0.001 %
	Minimum absolute height	3.0E3
	Min ratio of peak top/edge	1.7
	Peak duration range	0-2 min
Isotope grouping	m/z tolerance	0-10 ppm
	RT tolerance	0.2 min
	Maximum charge	3
Alignment	Algorithm	Join aligner
	m/z tolerance	0-20 ppm
	Weight for m/z	75%
	RT tolerance	0.2 min
	Weight for RT	25%

Table S3. A list of annotated compounds in *A. keratiniphila* DPA04.

No.	R_t (min)	Compound name	Adduct	Exact mass	Molecular formula	MS/MS Fragmentation Product Ions	Chemical class	Culturing medium
1	0.40	Sulfurol[a]	M+H	143.040486	C_6H_9NOS	99.026, 112.0212, 113.0295, 126.0372	Thiazole derivatives	M1
2	0.42	Maltotriose[a]	M+Na	504.169040	$C_{18}H_{32}O_{16}$	347.0966, 365.1064, 467.1388, 509.1493	Sugars	ISP-2, SNA, R2YE, Cz
3	0.43	Raffinose[b]	M+Cl	504.169040	$C_{18}H_{32}O_{16}$	89.024, 101.0243, 221.0656, 383.1184	Sugars	ISP-2, SNA, R2YE, Cz
4	0.87	Ethylmaltol[a]	M+H	140.047345	$C_7H_8O_3$	55.0166, 71.0113, 97.0263, 126.0289	Sugars	ISP-2, M1
5	1.20	5-Chlorosalicylamide[b]	M-H	171.008707	$C_7H_6ClNO_2$	126.9953, 134.0238, 151.9909, 170.0011	Benzamides	ISP-2, M1
6	2.83	Methyl-2,3,5-Trihydroxytetradecanoate[b]	M-H	290.209325	$C_{15}H_{30}O_5$	59.0145, 157.1232, 229.1818, 271.1915	hydroxylated fatty acid esters	R2YE, Cz
7	2.90	20-Hydroxyrifamycin S[c]	M-H	711.289094	$C_{37}H_{45}NO_{13}$	123.045, 180.1031, 221.0471, 253.0712	Macrocyclic lactams	ISP-2, M1
8	3.07	Aleuritic acid[b]	M-H	304.224975	$C_{16}H_{32}O_5$	59.014, 73.0301, 285.2069, 301.201	Hydroxylated fatty acids	R2YE, Cz
9	3.13	Tirandamycin F[c]	M-H	405.178754	$C_{21}H_{27}NO_7$	58.0305, 123.0817, 193.0505, 345.1345	Aminoglycosides	ISP-2, M1
10	3.21	Rifamycin S[b]	M-H	695.294179	$C_{37}H_{45}NO_{12}$	123.0453, 153.0559, 180.1034, 221.0462	Macrocyclic lactams	ISP-2, M1
11	3.35	Methyl-aleuritinat[b]	M-H	318.240625	$C_{17}H_{34}O_5$	59.0137, 257.1744, 299.2213, 315.2157	Hydroxylated fatty acid esters	R2YE, Cz
12	3.63	Epothilone N[d]	M+H	545.221404	$C_{26}H_{40}ClNO_7S$	281.0618, 288.156, 446.1793, 514.2032	Macrolides	ISP-2
13	3.85	3,15-dihydroxypentadecanoic acid [b]	M-H	274.214410	$C_{15}H_{30}O_4$	59.0146, 87.045, 255.1963, 257.1759	Hydroxy fatty acids	R2YE, Cz
14	3.85	Rifamycin O[b]	M-H	753.299659	$C_{39}H_{47}NO_{14}$	119.0862, 154.0744, 163.0772, 192.1029	Macrocyclic lactams	ISP-2, M1
15	3.91	sn-1-lyso-2-16:0-MGDG/ 2-Hexadecanoyl-3-*O*-*β*-D-galactosyl-sn-glycerol[b]	M-H	492.329835	$C_{25}H_{48}O_9$	59.0143, 71.0139, 89.0244, 101.0239	Galactoglycerolipids	ISP-2, M1
16	3.98	Microsclerodermin A[d]	M+H	994.428382	$C_{47}H_{62}N_8O_{16}$	95.0855, 177.0903, 269.1024, 299.1141	Cyclic peptides	ISP-2
17	4.03	Antimycin A20 [c]	M+H	506.226433	$C_{25}H_{34}N_2O_9$	135.0767, 179.0778, 191.103, 205.093	Depsipeptides	ISP-2, M1

18	4.07	15-hydroxy-2,15-dimethyl-3-{[3,4,5-trihydroxy-6-(hydroxymethyl)oxan-2-yl]oxy}hexadecanoic acid [b]	M-H	478.31418	$C_{24}H_{46}O_9$	59.0145, 71.0143, 89.0246, 101.0242	Hydroxylated fatty acid esters	ISP-2, M1
19	4.13	AK_1 [d]	M+H	741.456432	$C_{41}H_{63}N_3O_9$	73.056, 101.0861, 155.1322, 548.415	Linear polyketides	ISP-2, M1
20	4.41	N-demethyl ECO-0501[d]	M+H	822.477896	$C_{45}H_{66}N_4O_{10}$	86.0714, 141.1259, 192.0659, 629.4438	Linear polyketides	ISP-2, M1
21	4.48	ECO-0501[d]	M+H	836.493545	$C_{46}H_{68}N_4O_{10}$	101.0949, 155.1419, 348.3016, 643.4592	Linear polyketides	ISP-2, M1
22	4.87	LysoPE (16:0/0:0)[a]	M-H	453.285541	$C_{21}H_{44}NO_7P$	78.9594, 140.0119, 196.0377, 255.2333	Phospholipids	SNA, R2YE, Cz
23	4.97	LysoPI (16:0/0:0) [a]	M-H	572.296168	$C_{25}H_{49}O_{12}P$	152.9957, 241.0123, 255.2333, 315.0485	Glycerophospholipids	SNA, R2YE, Cz
24	5.02	LysoPE 17:0/ 1-Heptadecanoyl-glycerophosphoethanolamine[b]	M-H	467.301192	$C_{22}H_{46}NO_7P$	78.9596, 140.0118, 196.0377, 269.2489	Glycerophospholipids	SNA, R2YE, Cz
25	5.19	[1-Hexanoyloxy-3-[3,4,5-trihydroxy-6-(hydroxymethyl)oxan-2-yl]oxypropan-2-yl] decanoate[b]	M-H	506.309100	$C_{25}H_{46}O_{10}$	59.0145, 75.0092, 113.0241, 255.2332	Fatty acid esters	ISP-2, M1
26	5.39	[1-Hexanoyloxy-3-[3,4,5-trihydroxy-6-(hydroxymethyl)oxan-2-yl]oxypropan-2-yl] undecanoate[b]	M-H	520.324750	$C_{26}H_{48}O_{10}$	71.0146, 113.0247, 249.0614, 269.2487	Fatty acid esters	ISP-2, M1
27	5.51	Citroflex 4 [b]	M+H	360.214805	$C_{18}H_{32}O_7$	57.07, 111.0073, 129.0179, 139.0021	Organooxygen compounds	ISP-2, M1, SNA, R2YE, Cz
28	5.57	Azathymine [b]	M+Na	127.038177	$C_4H_5N_3O_2$	65.0388, 66.0422, 94.0369, 122.0314	Pyrimidinones	ISP-2, M1, SNA, R2YE, Cz
29	5.65	Gageostatin A [c]	M-H	1039.678053	$C_{52}H_{93}N_7O_{14}$	323.1602, 339.2038, 452.2873, 696.4917	Linear lipopeptides	M1
30	5.90	Surfactin A [a]	M+H	1007.651838	$C_{51}H_{89}N_7O_{13}$	86.0965, 199.1804, 227.1752, 324.2166	Cyclic lipopeptides	M1
31	6.08	Surfactin B [a]	M+H	1021.667488	$C_{52}H_{91}N_7O_{13}$	86.097, 199.1813, 227.176, 338.2341	Cyclic lipopeptides	M1, Cz
32	6.67	PA(22:5(7Z,10Z,13Z,16Z,19Z)/22:5(7Z,10Z,13Z,16Z,19Z)) [b]	M-H	796.504308	$C_{47}H_{73}O_8P$	78.9596, 241.0124, 241.2181, 255.2336	Phospholipids	SNA, R2YE

33	6.72	Nocardimicin R [e]	M+H	869.551395	$C_{47}H_{75}N_5O_{10}$	57.0709, 59.05, 188.0922, 567.4985	Siderophores	ISP-2, SNA, R2YE, Cz
34	6.72	1,4-didodecyl-2,3-dihydroxybutanedioate [b]	M+H	486.392040	$C_{28}H_{54}O_6$	55.055, 57.0344, 75.0447, 83.0862	Fatty acid esters	SNA, Cz
35	6.74	PI(32:0)/ 1,2-dihexadecanoyl-sn-glycero-3-phospho-D-myo-inositol[b]	M-H	810.525832	$C_{41}H_{79}O_{13}P$	78.9596, 241.0124, 241.2181, 255.2336	Glycerophospholipids	SNA, R2YE, Cz
36	6.83	LysoPI(16:0/0:0)/ 1-*O*-(1-O-hexadecanoyl-2-*O*-heptadecanoyl-sn-glycero-3-phosphono)-1D-myo-inositol[b]	M-H	824.541483	$C_{42}H_{81}O_{13}P$	152.9953, 241.0116, 255.2329, 269.2483	Glycerophospholipids	SNA, R2YE, Cz
37	6.83	PS(15:0/16:0)/ 1,2-Dipalmitoylglycerophosphorylserine [b]	M-H	721.489387	$C_{37}H_{72}NO_{10}P$	211.2068, 255.2338, 257.2123, 271.2279	Phospholipids	SNA, R2YE
38	7.21	Glucolipsin A[b]	M+NH4	916.6487	C50H92O14	59.042, 127.0445, 279.2739	Fatty acid glycosides	M1,ISP-2,SNA
39	7.36	Glucolipsin B[b]	M+NH4	920.6685	C49H90O14	57.0708, 85.0293, 127.0399, 265.2539	Fatty acid glycosides	M1,ISP-2,SNA,R2YE,Cz
40	7.37	N, N-Dibutyloleamide [b]	M+H	393.397064	$C_{26}H_{51}NO$	57.0708, 134.097, 135.1, 322.2542	Amides	ISP-2, M1, SNA, R2YE, Cz
41	7.48	Lipocarbazole A1 [d]	M+H	429.303164	$C_{30}H_{39}NO$	288.1373, 359.2242, 360.2276, 431.3142	Carbazoles	ISP-2, M1, SNA, R2YE, Cz
42	7.48	P,P-Dioctyldiphenylamine[b]	M+H	393.339549	$C_{28}H_{43}N$	57.0721, 134.0986, 135.1018, 323.2635	Aromatic amines	ISP-2, M1, SNA, R2YE, Cz
43	7.48	1-Heptyl-2-methyl-9H-carbazol-3-ol [d]	M+H	295.193614	$C_{20}H_{25}NO$	57.0715, 210.0932, 225.1173, 297.2068	Carbazoles	ISP-2, M1, SNA, R2YE, Cz
44	7.56	[(2S)-3-[(2R,3R,4S,5R,6R)-6-(formyloxymethyl)-3,4,5-trihydroxyoxan-2-yl]oxy-2-hexadecanoyloxypropyl] hexadecanoate [b]	M-H	758.554415	$C_{42}H_{78}O_{11}$	113.0242, 255.2325, 269.2478, 283.2637	Fatty acid esters	ISP-2, M1
45	7.67	*α*-GlcA-DAG (C18:0/C16:0)/ 3-*α*-D-glucuronosyl-2-palmitoyl-1-stearoyl-sn-glycerol [b]	M-H	772.570065	$C_{43}H_{80}O_{11}$	113.0233, 255.232, 269.2465, 283.2629	Fatty acid esters	ISP-2, M1

[a]Indicates metabolite annotation according to the Global Natural Product Social Molecular Networking (GNPS), [b]Indicates metabolite annotation according to Sirius, [c]Indicates metabolite annotation according to The Natural Products Atlas (NPAtlas), [d] indicates metabolite annotation according to Moldiscovery, and [e]Indicates metabolite annotation according to Reaxys.

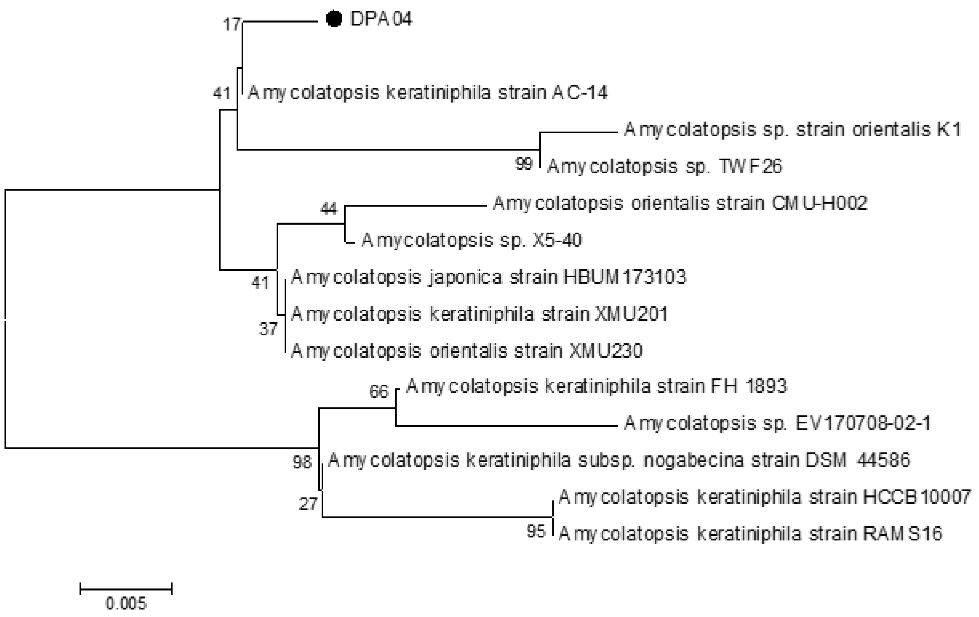

Figure S1. A neighbor-joining phylogenetic tree based on 16s rRNA sequences. Dark circle represents bacterial isolate used in this study DPA04; bootstrap values are indicated at each node.

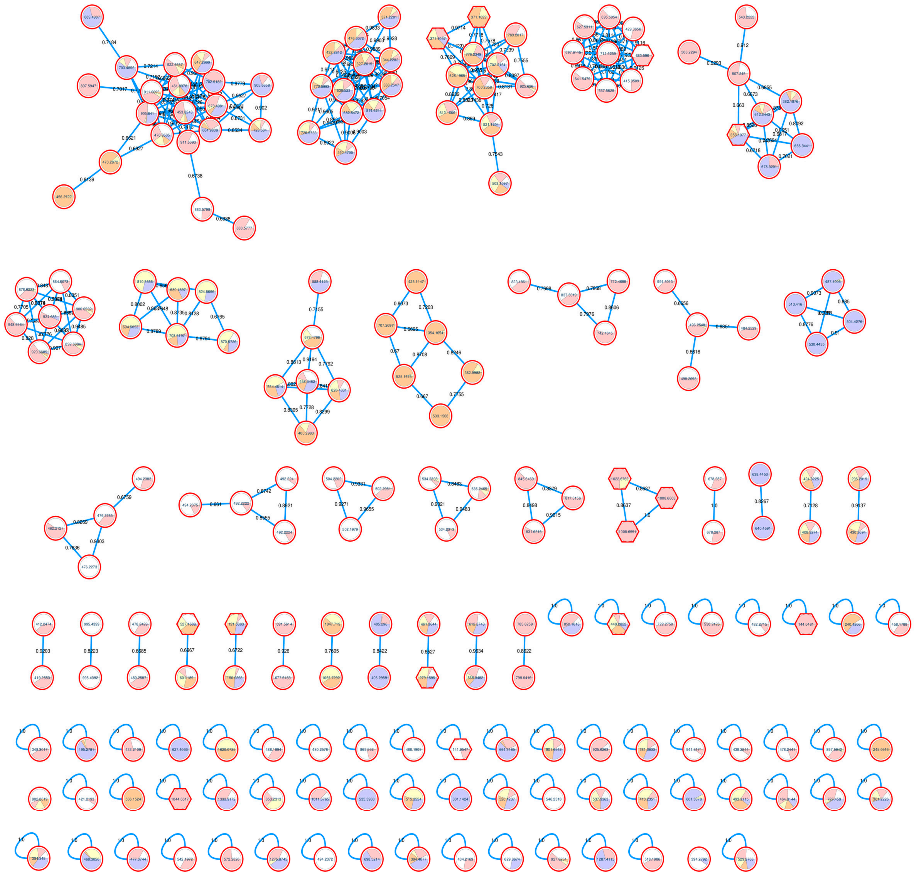

Figure S2. Molecular network of positive ion mode data where the node color represents culture media as follows: white= ISP-2, red= M1, purple= SNA, orange= R2YE, and yellow= Czapek.

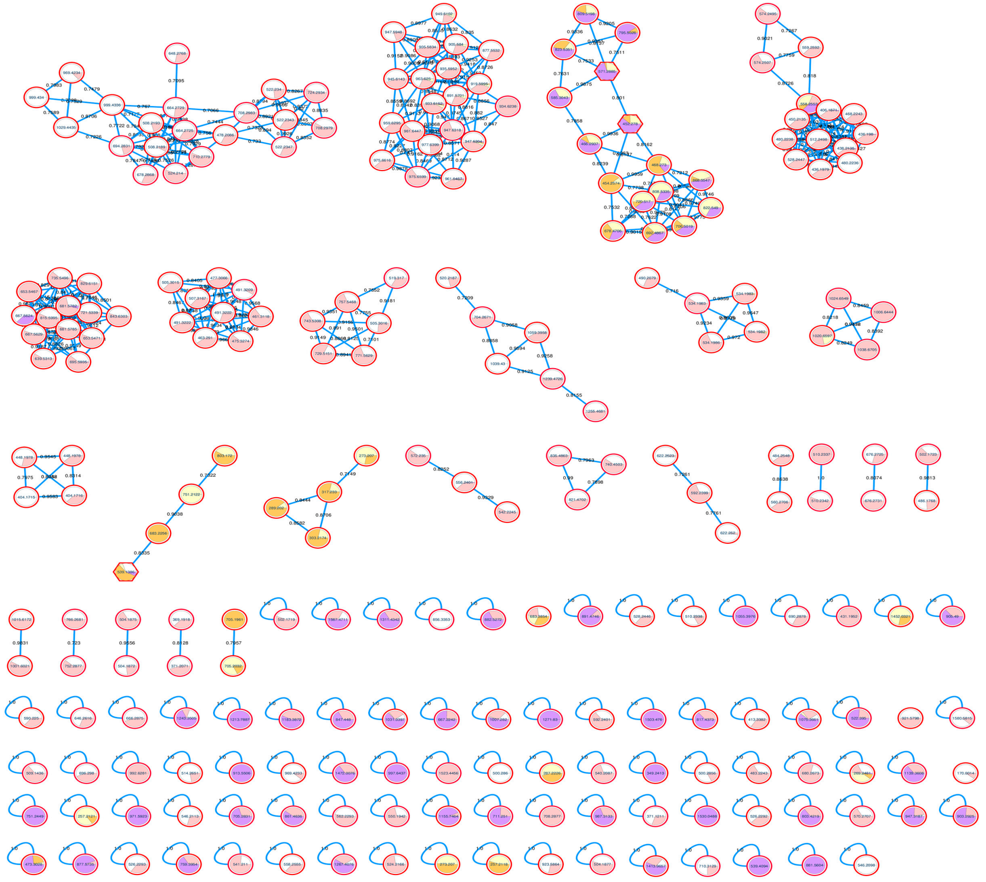

Figure S3. Molecular network of negative mode data where the node color represents culturing media as follows: white= ISP-2, red= M1, purple= SNA, orange= R2YE and yellow= Czapek.

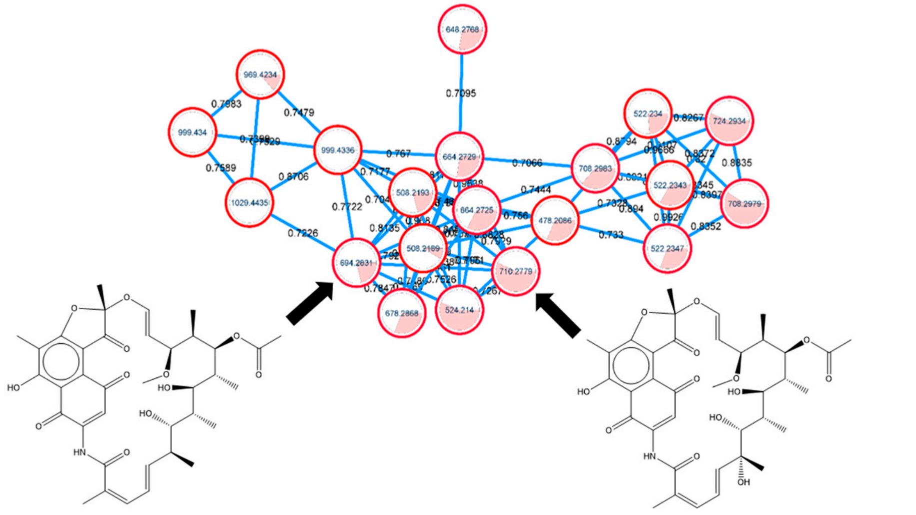

Rifamycin S

20-hydroxyrifamycin S

Figure S4. Rifamycins spectral family with annotated metabolites where the node color represents culturing media: white= ISP-2, red= M1. The edge label represents the cosine score.
